# The Cellular Distribution and Ser262 Phosphorylation of Tau Protein Are Regulated by BDNF In Vitro

**DOI:** 10.1371/journal.pone.0091793

**Published:** 2014-03-11

**Authors:** Qian Chen, Zhou Zhou, Lei Zhang, Shangcheng Xu, Chunhai Chen, Zhengping Yu

**Affiliations:** Department of Occupational Health, Third Military Medical University, Chongqing, China; Alexander Fleming Biomedical Sciences Research Center, Greece

## Abstract

The brain-enriched microtubule-associated protein tau, a critical regulator of cytoskeletal dynamics, forms insoluble aggregates in a number of neurodegenerative diseases termed tauopathies, including Alzheimer's disease (AD). Hyperphosphorylation of tau protein is an important mechanism for aggregation, so many studies on the pathogenesis of AD and other tauopathies have focused on regulation of tau phosphorylation by kinases and phosphatases. Less studied are mechanisms of tau transcriptional and post-transcriptional regulation by extracellular signals such as BDNF and how such changes alter neuronal function. Previously, we reported that tau is required for morphological plasticity induced by BDNF. Here, we further explore tau modification during BDNF-induced changes in neuronal cell morphology. In undifferentiated SH-SY5Y cells lacking neurites, tau formed a sphere within the soma as revealed by immunocytochemistry. In contrast, tau was enriched in the neurites and sparse in the soma of SH-SY5Y cells induced to differentiate by retinoic acid (RA). Treatment with RA also increased total tau protein levels but decreased expression of tau phosphorylated at Ser262 as determined by Western blot. Both effects were further enhanced by subsequent BDNF treatment. Upregulation of tau protein and downregulation of p-Ser262 tau were correlated with total neurite length (R = .94 and R = −.98, respectively). When primary E18 hippocampal neurons were treated with nocodazole, a blocker of microtubule polymerization, nascent neurites were lost and tau shifted to the soma. This process of retrograde tau movement away from neurites was reversed by BDNF. These results indicate that tau is redistributed to neurites and dephosphorylated during RA- and BDNF-mediated differentiation.

## Introduction

Dysregulation of the protein tau is implicated in the pathogenesis Alzheimer's disease (AD) and many other neurodegenerative disease [1]. Tau is a microtubule (MT)-associated protein that acts to regulate cytoskeletal dynamics by transient MT binding and unbinding, a process controlled in part by tau phosphorylation [2]. The aberrant assembly of tau into filaments is the primary histopathological hallmark of several human neurodegenerative diseases, collectively known as tauopathies [3]. Thus, describing the signaling pathways controlling tau expression, subcellular distribution, and phosphorylation status could lead to the development of treatments for AD and other tauopathies.

Tau is essential for the maintenance of polarity and for the survival of mature neurons [4]. The neurophysiological functions of tau protein depend mainly on its subcellular distribution and phosphorylation status [5], [6]. Overexpression of tau appears to destabilize the MT, but it is unclear whether this phenomenon is always pathogenic and whether it results in loss-of-function or gain-of-function [7]. Tau protein loss-of-function would disrupt cytoskeletal stability and may explain tau-related pathological progression [8]. However, recent finding also support the notion that tau protein gain-of-function has a significant role in AD pathogenesis, either alone or in the presence of other risk factors such as Aβ [9]. Whether pathogenic changes in tau result in protein loss-of-function or toxic gain-of-function largely depends on specific modifications in protein conformation, distribution, and phosphorylation [7].

Some familial neurodegenerative diseases have been linked to tau gene (MAPT) mutations that lead to alternative splicing, changes in phosphorylation state, reduced affinity for tubulin, and (or) enhanced capacity for self-association into filaments and aggregates [10]. The wild type tau protein has over 85 phosphorylation sites as identified by mass spectrometry, including Ser199/202, Ser213, Ser262, Ser396, and Ser404. Phosphorylation of these sites differentiates tau-dependent neuronal toxicity and dysfunction [11]. A major focus of current AD research is the development of drugs that decrease either tau phosphorylation or total protein expression [1], [12].

In addition to MT stabilization, tau protein interacts with many signaling pathways to regulate the intracellular trafficking of organelles and molecule involved in presynaptic and postsynaptic function [7], [13]. Tau protein participates in the morphological plasticity induced by brain-derived neurotrophic factor (BDNF) [14], a ubiquitous neurotrophin critical for neuronal differentiation as well as synaptic plasticity and synaptogenesis. In addition, BDNF is an effective neuroprotectant in many models of neurological disease and a promising therapy for the treatment of AD and other neurological and psychological disorders [15], [16], [17]. Here, we demonstrate that the cellular distribution of tau and its phosphorylation at Ser262 are correlated with neurite outgrowth induced by RA and BDNF. Specifically, Ser262 dephosphorylation is associated with a shift from accumulation in the soma to co-distribution with tubulin in neurites.

## Materials and Methods

### Cell cultures

Human SH-SY5Y neuroblastoma cells from ATCC were cultured in Dulbecco's modified Eagle's medium / F12 medium (Invitrogen, Carlsbad, CA, USA) with 10% heat-inactivated fetal bovine serum (HyClone, Logan, UT, USA), 1% v / v penicillin / streptomycin (Sigma-Aldrich, St Louis, MO, USA) in a 5% CO_2_ humidified atmosphere at 37°C. Cells were grown with or without all tans-retinoid acid (RA) for 5 days before treatment.

Primary hippocampal neurons were prepared from E18 rat fetuses as previously described [18]. Similarly, hippocampi dissected from brains of the fetuses using fine forceps was treated with 0.25% trypsin for 20 min and triturated with a Pasteur pipette. The cell suspensions were plated on poly-L-lysine-coated coverslips and maintained in glial-conditioned medium (Neurobasal, 2% B27 supplement, 1% L-glutamine) at 37°C under 5% CO_2_ until use. Neurobasal and B27 supplement were purchased from Invitrogen (Carlsbad, CA).

### Ethics statement

All experiments involving the use of animals were performed in accordance with the European Communities Council Directive of 24 November 1986 (86/609/EEC) and animal care guidelines approved by the Institute of Experimental Medicine ASCR Animal Care Committee. The protocol was approved by the Committee on the Ethics of Animal Experiments of the Third Military Medical University. All efforts were made to minimize animal suffering and to reduce the number of animals used.

### Pharmacological reagents

BDNF, RA, taxol and nocodazole were purchased from Sigma–Aldrich (St. Louis, MO). BDNF was dissolved in sterilized water at 200 nM. Taxol and nocodazole were dissolved in DMSO (Sigma–Aldrich, St. Louis, MO) at 1 mM and 20 mM separately. Taxol and nocodazole solution were stored at 4°C and BDNF solution was stored at −20°C until use.

Primary cultured hippocampal neurons were pre-incubated for 2 h with taxol or nocodazole at the following concentration before BDNF incubation: taxol (10 nM), nocodazole (100 nM for 3 day in vitro (DIV) neurons, 500 nM for 13DIV neurons.

### Western blot and antibodies

Western blot was performed as previously described [14]. Briefly, cells were washed with ice-cold PBS and scraped in RIPA lysis buffer including protease inhibitors and phosphorytase inhibitors (Roche,Indianapolis,IN). After electrophoresis, proteins were transferred to polyvinylidene difluoride (PVDF) membranes (Millipore. Billerica, MA). Non-specific binding was blocked by incubation overnight at 4°C with 5% non-fat dry milk in PBS. Membranes were then incubated in primary antibodies for 12 h or overnight at 4°C. The following primary antibodies were used separately: mouse monoclonal Tau5, rabbit polyclonal phospho-Tau (Ser262) (Invitrogen, Camarillo, CA), mouse monoclonal anti-β-actin antibody (Sigma, St. Louis, MO). Membranes were washed three times with the TBST and then incubated with the Fluor-conjugated secondary antibody (LI-COR, Lincoln, NE). After final washes with PBS, the signal was then detected and quantified with Odyssey infrared imaging system (LI-COR, Lincoln, NE). Loading controls were performed with anti-β-actin antibody.

### Immunocytochemistry and Immunofluorescence intensity measurement

Immunocytochemistry was performed as previously described [19]. Cells were rinsed once with PBS and fixed by the rapid addition of 100% methanol (4°C). Fixed cells were incubated overnight in blocking buffer (PBS containing 3% BSA, 0.1% Triton X-100, and 1% horse serum). Cells were incubated first with Tau5 antibody, then Rhodamine-conjugated donkey anti-mouse secondary antibody (Invitrogen, Camarillo, CA), followed by FITC-conjugated mouse monoclonal α-tubulin antibody (Invitrogen, Camarillo, CA). All incubations were for 1 h at room temperature, followed by four 15-min washes in blocking buffer. Nuclei were counterstained with Hoechst 33342 DNA stain. Coverslips were mounted on glass slides using antifade mounting medium. Images were obtained using Leica confocal laser scanning microscope (63×, TCS SP5, Germany). Images were 4 Kalman averages of the same plane in the *z*-axis. Images were processed using Adobe Photoshop 3CS (San Jose, CA). Immunofluorecence intensity measurement was performed with the aid of LAS-AF software (Leica, Germany). Immunofluorescence of tau and α-tubulin in the soma part of each neuron was analyzed. The ratio of average intensity of tau and tubulin was recorded. Data were shown as mean ratio ± SD (n = 40).

### Neurite length analysis

Neurite length of hippocampal neurons was measured as previously described [14]. Data were shown as mean neurite length ± SD (n = 30). For quantitative analysis of the morphological changes of SH-SY5Y cells induced by pharmacological reagents, we estimated the total neurite length of SH-SY5Y cells stained by immunocytochemistry. First, we labeled fixed SH-SY5Y cells with mouse monoclonal Tau 5 antibody and FITC-conjugated α-tubulin antibody by immunocytochemistry as described above. Then, we obtained images of the neurons using Leica confocal laser scanning microscope (40×, TCS SP5, Germany), and measured the length of each process on the fluorescence images to estimate the length of neurites in the cells with LSM image browser (Leica, Germany). In this analysis, the measured region on the protrusions was from the tip to the base of the process estimated from the outline of nuclei. After the measurement, we recorded all the lengths of processes from a neuron, and also the number of neurites of each neuron. If neurons in these fluorescence images overlapped with neighboring neurons, they were excluded from this analysis. All data were shown as mean total neurite length ± SD (n = 100).

### Statistic analysis

All the quantitative data were obtained from three independent experiments and presented as the mean ± SD. Statistical significances of differences between two groups were determined by using unpaired Student's t test. All P values were two sided, and P<0.05 was considered as statistical significance.

## Results

### Changes in tau distribution during retinoic acid (RA)-induced differentiation of SH-SY5Y cells

**Figure 1:**
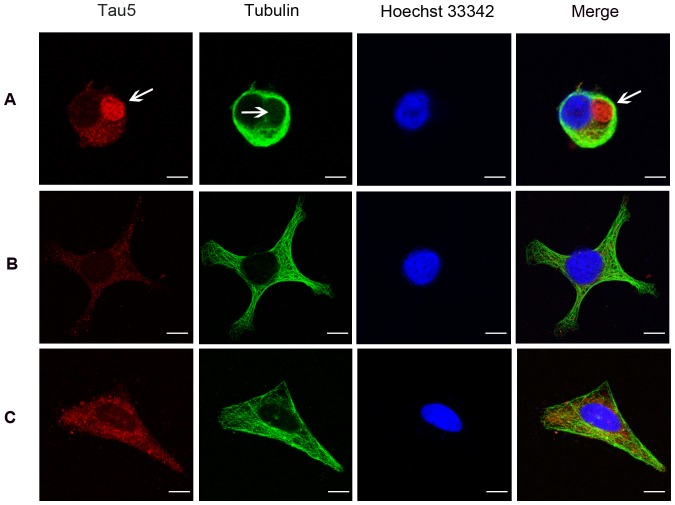
Tau protein distribution in undifferentiated and RA-differentiated SH-SY5Y cells. (A) Tau protein formed a spherical structure (arrow in the left panel) in the soma of some undifferentiated SH-SY5Y cells. No α-tubulin staining was detected in the same area (arrow in the tubulin panel). Co-immunostaining for α-tubulin and tau showed no detectable overlap (arrow in the right panel). (B) Tau protein distributed along the cytoskeleton of undifferentiated SH-SY5Y cells with dendrites. (C) RA treatment shifts the distribution of tau protein away from the soma and into neurites (Bar = 10 μm).

**Figure 2:**
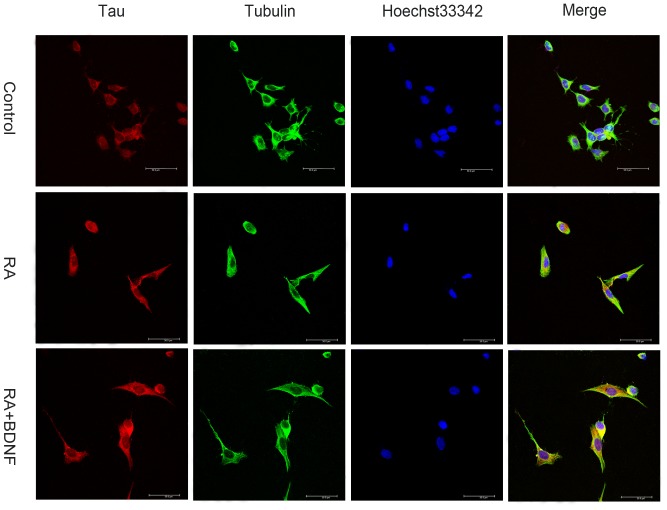
BDNF increased tau protein expression in RA-differentiated SH-SY5Y cells. RA treatment induced neurite formation/elongation in SH-SY5Y cells and increased tau expression. Subsequent BDNF treatment further increased neurite number/length and tau protein expression (Bar = 50 μm).

**Figure 3:**
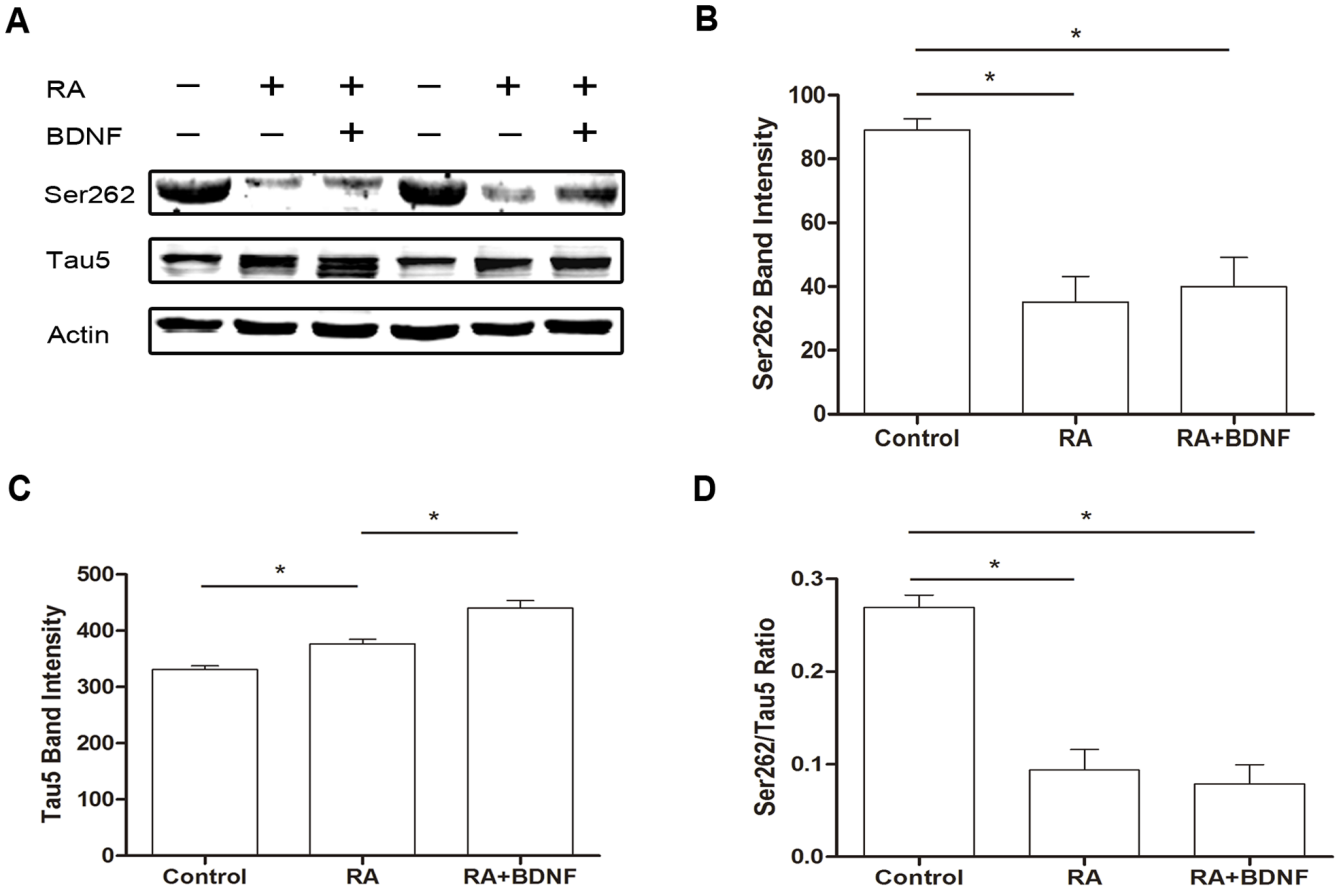
Tau protein expression and phosphorylation at Ser262 in SH-SY5Y cells after RA and BDNF stimulation. (A) RA increased total tau protein expression and Ser262 dephosphorylated tau in SH-SY5Y cells. BDNF further increased tau protein. (B) Quantification of pSer262 tau. (C) Quantification of tau protein expression. (D) Fraction of total tau phosphorylated at Ser262. Each experiment were repeated for 3 times (* p<0.05).

Changes in the subcellular distribution and conformation of tau during the progression of tauopathies have been studied extensively in an attempt to understand the process of neurofibrillary tangle (NFT) formation, the aberrant conformation of tau that is a hallmark of these diseases [20]. To this end, SH-SY5Y cells were differentiated with RA for five days before BDNF treatment for 24 h. Then cells were harvested for Western blotting or fixed for immunohistochemistry. In this study, we examined the distribution of tau and α-tubulin in undifferentiated and RA-differentiated SH-SY5Y cells by immunocytochemical staining using mouse monoclonal antibodies against tau5 and α-tubulin. In some undifferentiated SH-SY5Y cells with no protrusions (neurites), immunostaining revealed that tau formed a spherical inclusion (arrow in the left panel) in the soma next to the nucleus (Figure 1A). Co-immunostaining for α-tubulin and tau showed no detectable overlap (Figure 1A, arrow in the right panel) in these undifferentiated cells, with most α-tubulin localized to the soma periphery. Thus, in undifferentiated SH-SY5Y cells, tau is not associated with MT (off-the-MT status). In undifferentiated cells with neurites, however, tau protein was scattered throughout the neurites with little in the soma, and immunostaining overlapped extensively with that of α-tubulin (Figure 1B). Induction of differentiation using RA resulted in neurite formation, upregulation of tau and a shift in tau distribution from soma to neurites (Figure 1C). The spherical structure seen in undifferentiated cells appeared similar to tau inclusions in AD. In addition to roles in neural development, BDNF influences learning and memory by regulating synaptic plasticity [21], [22]. Here we applied BDNF to RA-differentiated SH-SY5Y cells to examine possible modifications in tau expression, distribution, and structure. As revealed by immonocytochemistry, tau protein levels were elevated in RA-treated cells compared to untreated controls (Figure 2A, B), while subsequent application of BDNF further increased tau expression. In contrast, these treatments did not appear to alter α-tubulin expression or distribution. The increase of tau protein level was also confirmed by western blot (Figure 3A, C).

### Correlation between tau protein expression and neurite growth

These immunostaining studies in differentiating SH-SY5Y cells suggests that tau protein expression is upregulated and shifted away from the soma during neurite formation, possibly due to reduced phosphorylation and enhanced association with α-tubulin. Tau protein phosphorylation at Ser262 greatly influences the binding of tau to MTs [23]. In our previous study, regulation of Ser262 phosphorylation appeared independent of phosphorylation at other sites, finding also reported by others [24]. In the current study, phosphorylation at Ser262 was decreased by about 60% following RA treatment compared to untreated cells (Figure 3A, C). The difference between RA-treated and RA+BDNF-treated cells did not reach statistical significance (Figure 3C, p = 0.59 compared to RA). Correcting for changes in total tau expression, the reduction induced by BDNF was larger, though still not significant (Figure 3D, p = 0.31).

**Figure 4:**
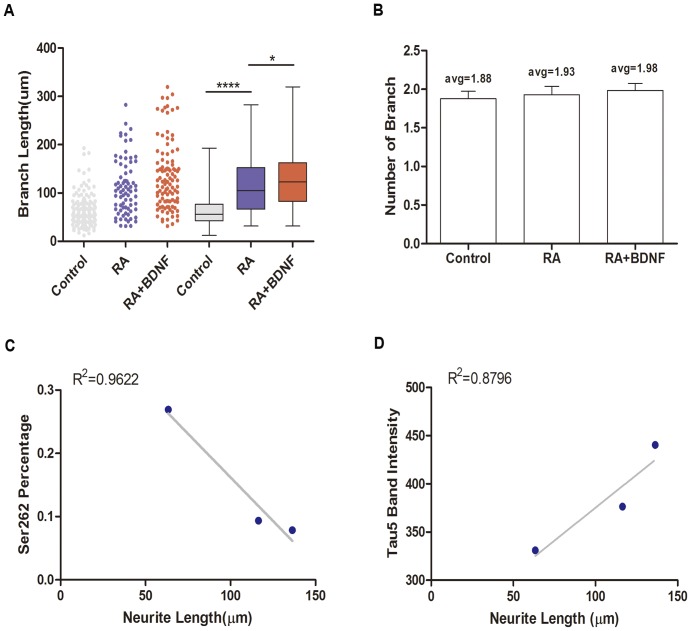
Neurite growth in SH-SY5Y cells after application of RA and BDNF. (A) Branch length of SH-SY5Y cells. Branch length was markedly increased by RA stimulation. The application of BDNF further increased branch length (*p<0.05, **** p<0.0001). (B) Number of branches per cell. The average of branch number was not altered by these treatments (1.88 for controls, 1.93 for RA-treated, and 1.98 for RA+ BDNF-treated cells). (C) Plot of average neurite length versus p262 tau. Linear regression revealed a negative correlation between neurite length and p262 tau expression (R^2^ = 0.8796). (D) Plot of average neurite length versus total tau. Linear regression revealed a positive correlation between neurite length and total tau expression (R^2^ = 0.9622).

Tau binding to MTs is a critical regulator of the balance between MT polymerization and depolymerization, and thus a major influence on cytoskeletal structure and cell morphology [25], [26]. Neurite outgrowth depends on local MT polymerization, and indeed MT immunostaining was largely restricted to neurites in differentiated SH-SY5Y cells. To establish a quantitative relationship between total tau expression, Ser262 phosphorylation status, and neurite elongation during differentiation, we measured total neurite length of RA- or RA+BDNF-treated cells using Leica LSM software and plotted these values against protein band density measured by Western blot. RA treatment increased average neurite length from 63.40 μm to 116.45 μm (Figure 4A, p<0.0001), while BDNF further stimulated neurite elongation to 136.37 μm (Figure 4A, p<0.05 vs. RA alone). In contrast to neurite length, neither RA alone nor RA+BDNF significantly increase the number of branch per cell (Figure 4B). Linear regression revealed a negative correlation between average neurite length and Ser262 phosphorylation (R^2^ = 0.9622, Fig. 4C) and a positive correction between average neurite length and tau5 band density (R^2^ = 0.8796, Fig. 4D).

### Regulation of tau cellular distribution by nocodazole and taxol

**Figure 5:**
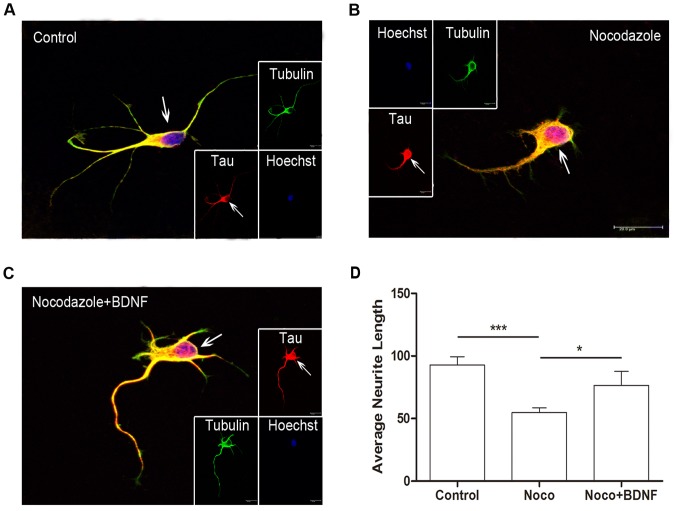
Tau protein distribution in DIV4 nocodazole-treated E18 primary hippocampal neurons. (A) Tau protein cellular distribution in control primary cultured hippocampal neurons. Arrow indicates the tau staining in the soma. (B) Example of nocodazole-damaged neuron. The tau protein signal (red) in the somatic compartment was much stronger than the Hoechst signal from the nuclei (blue), resulting in the merged purple in the soma as shown by arrow in the merged column. (C) Example of nocodazole-damaged neuron treated with BDNF. BDNF treatment decreased tau immunoreactivity in the soma compare to the nocodazole-treated group. Arrow indicates the tau staining in the soma (Bar = 20 μm). (D) Average neurite length of neurons in each group (*p<0.05, ***p<0.001).

**Figure 6:**
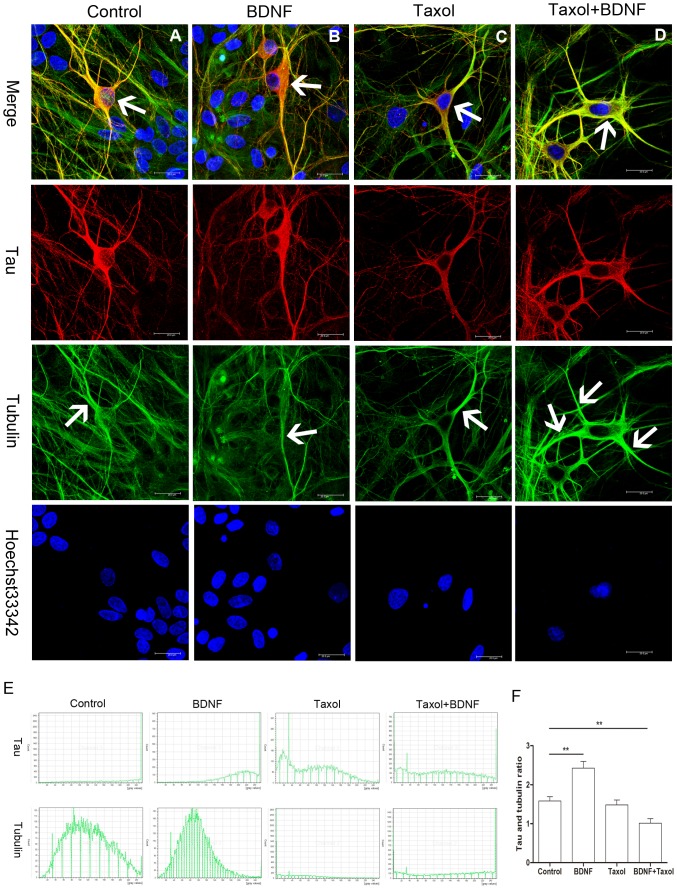
Tau protein distribution after BDNF, taxol and taxol+BDNF treatment. (A) Tau protein distribution in control E18 hippocampal neurons. Arrow indicates the tau staining in the soma in the merged column and the α-tubulin staining of neurite dendrites in the tubulin column separately. (B) BDNF increased tau protein immunostaining (arrow in the merged column) in the somata of DIV14 hippocampal neurons compare to control neurons. (C) Taxol stimulation did not appear to affect tau protein expression in the soma, but the dendrite tubulin signal (arrow in the tubulin column) was much stronger than in control and BDNF-treated neurons. (D) Tau protein immunoreactivity (arrow in the merged column) in the somata of taxol+BDNF-treated neurons was reduced compared to control, BDNF-treated, and taxol-treated neurons. A much stronger α-tubulin signal (arrow in the tubulin column) was observed and the co-localization of tau and α-tubulin immunostaining was strengthened as indicated by merged yellow (Bar = 20 μm). (E) Represent histogram of tau protein and α-tubulin intensity in the soma of neurons in each group. (F) Ratio of tau protein intensity against α-tubulin intensity in each group. The ratio in BDNF group is significantly higher than that of control group. Taxol + BDNF group got a lower ratio compared to control group (**p<0.01).

Increased phosphorylation and redistribution of tau may be a common response to neuronal stress [7], [27]. Tau shifts between the soma and neurites during neurite elongation and this shift is associated with Ser262 phosphorylation (Figures. 3, 4). We next examined if changes in MT polymerization and depolymerization alter tau distribution using the disruptor of polymerization nocodazole [28] and the polymerization facilitator taxol [29]. For these studies, we used DIV3 primary cultured hippocampal neurons from E18 Wistar rats. Nocodazole treatment for 24 h induced marked MT cytoskeletal damage as evidenced by neurite retraction (Figure 5). In previous reports, tau protein level was not altered by nocodazole treatment [14]. However, we observed intense tau immunoreactivity in the soma of nocodazole-treated neurons that obscured the blue emission from the Hoechst nuclear stain (Figure 5B, arrow). No α-tubulin staining was detected in this area (Figure 5B, arrow), in accord with results in SH-SY5Y cells (Fig. 1B). Treatment with BDNF partially reversed this effect of nocodazole, increasing tau and α-tubulin co-expression in neurites (Figure 5C, arrow). Compared to the control group, tau protein immunostaining was higher than that of α-tubulin in nocodazole- or nocodazole+BDNF-treated neurons. We then examined changes in tau distribution under conditions of enhanced MT polymerization (Figure 6). In accord with results from SH-SY5Y cells, BNDF increased tau protein level in the somatic compartment (as shown by arrow in the merged column) compared to control neurons (Figure 6A,B). On the other hand, taxol alone did not appear to alter soma tau protein expression but did enhance α-tubulin immunoreactivity (as shown by arrow in the tubulin column) in some dendrites (Figure 6C). Significantly, the enhanced tau protein signal in the soma (Figure 6D, arrow in the merged column) was not observed in hippocampal neurons treated with Taxol+BDNF, while the dendritic α-tubulin signal was enhanced (Figure 6D, arrow in the tubulin column). Tau and α-tubulin intensity in the soma part of each neuron was also analyzed (Figure 6E). Tau protein intensity was increased in soma as compared to the α-tubulin signal (Figure 6F).

**Figure 7:**
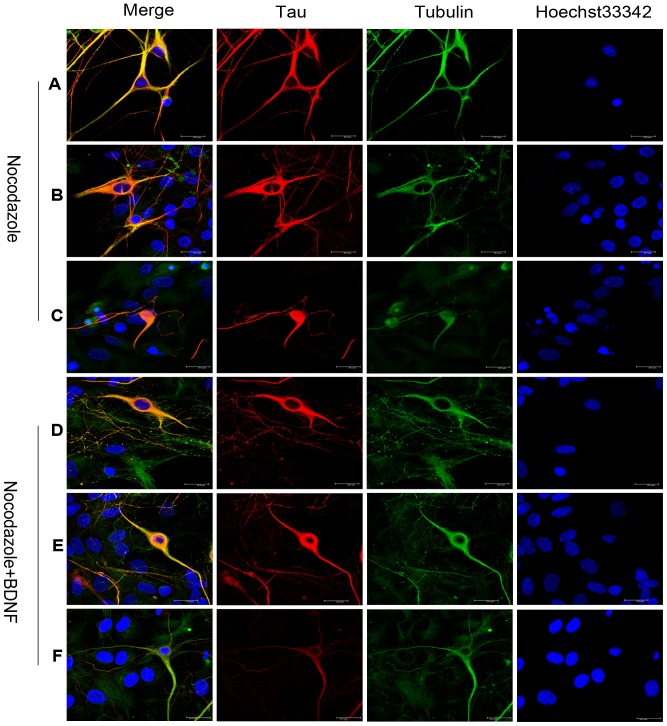
Effect of BDNF on tau distribution in nocodazole-treated hippocampal neurons. (A-C) DIV14 primary hippocampal neurons treated with nocodazole. (A) Tau protein distribution in lightly-damaged hippocampal neurons. (B) In more severely damaged neurons with only short dendrites, tau protein accumulated in the soma. (C) Heavily damaged neurons with higher tau protein immunoreactivity in the soma. (D-F) DIV14 primary hippocampal neurons treated with nocodazole+BDNF. Damaged hippocampal neurons with short dendrites show less soma tau localization after nocodazole+BDNF treatment compared to short-dendrite neurons treated with nocodazole alone. (E) Neurons with longer dendrites after nocodazole+BDNF treatment exhibiting stronger co-localization of tau and α-tubulin (yellow in merged image). (F) Neurons with extensive dendritic branching exhibiting strong tau and α-tubulin co-localization in dendrites with little tau expression in the soma after nocodazole+BDNF treatment (Bar = 20 μm).

The redistribution of tau protein was then examined in neurons with different degrees of nocodazole damage as indicated by the extent of neurite retraction. Compared to neurons with long dendrites (Figure 7A), tau immunostaining was much stronger in the soma of neurons with short dendrites (Figure 7B), and neurons with the shortest dendrites (those most severely affected by nocodazole) demonstrated the strongest tau immunostaining in the soma (Figure 7C). Thus, similar to SH-SY5Y cells (Fig. 4D), soma tau expression appeared to increase when neurites retracted. Also in accord with previous results, hippocampal neurons with shorter dendrites showed less somatic accumulation of tau following nocodazole+BDNF treatment compared to neurons treated with nocodazole alone (Figure 7D,E). Hippocampal neurons with normal morphology exhibited little tau immunostaining in the soma (Figure 7F).

Collectively, these results suggest that subcellular tau distribution is correlated with Ser262 phosphorylation status, dendritic length, and the balance between MT polymerization and depolymerization. Conditions that favor MT depolymerization and tau Ser262 phosphorylation are associated with accumulation of tau in spherical soma inclusions. Retinoic acid and BDNF appears to at least partially reverse these changes, suggesting a novel mechanism for the neuroprotective efficacy of these agents.

## Discussion

Brain-derived neurotrophic factor is neuroprotective against a variety of insults. In this study, we report that BDNF can increase tau protein expression in RA-differentiated SH-SY5Y cells (Figure 2,3) as well as primary cultured E18 hippocampal neurons [14] and shift the subcellular distribution away from the soma and toward the dendrites. Given the extensive interaction between tau protein, MTs, actin, and the plasma membrane, these changes in distribution may account for the effects of BDNF on dendritic complexity [30]. Furthermore, treatment with both BDNF and taxol redistributed tau protein from the somatic compartment to the dendritic compartment and enhanced the overlap of tau with MT (Figure 6), suggesting that MT polymerization (in this case taxol-induced) leads to tau redistribution and association with dendritic MTs. Thus, when there is demand for more MT stabilizer, as with taxol-treated neurons in our experiment (Figure 6), the upregulation of tau protein after BDNF stimulation may stabilize MTs. On the contrary, nocodazole-induced MT depolymerization may lead to neurons damage [31] associated with dendritic retraction and a shift in tau protein away from (shorter) dendrites and into the soma, where it accumulates (Figure 5,7). Accumulation of tau protein in the somatic compartment may be a common neuronal response to stressors such as serum deprivation, oxidative stress upon hydrogen peroxide treatment, or Aβ oligomer treatment [32]. The BDNF-mediated shift in tau may be a protective or reparative mechanism to alleviate the damage (Figure 5,7). Since BDNF and tau protein are critical factors both engaged in neuronal physiology activity and the development of neurodegenerative disease [33], [34], the meaning of tau protein up-regulation by BDNF should be carefully interpreted. Tau expression level is controlled at transcription level and at post-translation level. Experiment showed that the tau increase to nerve growth factor is due in part to changes in the mRNA levels [35]. Previous reports implicated that BDNF might enhance both translation from pre-existing tau mRNA and increase de novo synthesis as detected by real-time PCR and westernblot [14]. The increase of tau protein might also comes about by the impaired proteasomal degradation of tau [36] or regulation of tau stability directly or indirectly [37].The autoregulatory mechanism involving tau mRNA stability and the changes in tau expression and protein stability are clearly responding to the pools of the protein themselves, which might both coordinate the differentiation response of microtubule cytoskeleton [35]. According to our results and previous reports [14], we speculate that tau protein upregulation by RA+BDNF treatment serves to stabilize MTs and enhance cytoskeletal growth [38], [39], while under stress, increased tau expression may be a neuroprotective mechanism [40].

The spherical tau aggregates we observed in undifferentiated SH-SY5Y cells (Figure 1) may share similarities with AD-associated NFTs. In many tauopathies, tau is hyperphosphorylated and released from MT, ApoE, Src, and possibly other binding partners, resulting in loss of tau function [41], [42]. Previous reports also suggested that loss of tau function may lead to neurodegeneration [43]. Indeed, it is widely believed that the neurodegeneration associated with tauopathies results from hyperphosphorylation and sequestration of insoluble tau [7]. Other groups have shown that tau hyperphosphorylation resulting in short neuronal processes [44]. Mutations in the tau KXGS repeats (Ser262, 324, and 356) strongly inhibit the outgrowth of neurites, even though phosphorylation at these sites accounts for only a minor fraction of the total phosphate groups on tau. In our experiment, dephosphorylation of Ser262 was strongly related to neurite growth (Figure 4). This is consistent with a loss of tau function due to Ser262 phosphorylation, leading to aberrant changes in neuronal morphology, particularly loss of dendritic complexity, and ensuing neurodegeneration, possibly due to loss of tropic factor stimulation. Mutations in tau protein or age-related cellular stressors disrupt the interaction of tau with the cytoskeleton and (or) increase tau phosphorylation, leading to abnormal soma accumulation of unbound tau and loss of tau in dendrites [32], [45], [46]. As observed in our experiment, nocodazole caused dendritic regression and a shift of tau distribution from dendrite to soma (Figure 5,7), a process that is believed to be a critical pathogenic step in tauopathies [3].

Our data also highlight BDNF signaling as a feasible target for drugs against tauopathies [47]. Inhibition of tau synthesis by transfection of oligonucleotides resulted in decreased tau protein levels and significantly shorter cellular processes [48]. Our data indicate that tau expression correlates with neurite growth, at least in culture (Figure 3). Our previous report demonstrated that tau protein upregulation accompanied neurite outgrowth in BDNF-treated hippocampal neurons. Here we show that this upregulation and subcellular shift in tau expression is strongly correlated with dephosphorylation at Ser262 (Figure 3), further supporting the notion that phosphorylation/dephosphorylation status is a major factor influencing neuronal polarity. Future studies are required to elucidate the signaling pathways linking BDNF and tau dephosphorylation.

The BDNF signaling cascade, including BDNF, TrkB, and downstream kinases and phosphatases are targets for drugs modifying tau expression, phosphorylation, and cellular distribution. Indeed, such treatments could benefit a broad spectrum of tauopathies, including AD [49], [50]. Moreover, RA is commonly used to induce the differentiation of neuroblastoma cells [51]. Our findings suggest an important role for RA in the regulation of tau expression and dephosphorylation at Ser262 (even in the absence of BDNF) (Figure 2,3). Alternatively, a previous study reported RA-induced tau phosphorylation at all examined sites, including Ser199, Ser202, Thr-205, Ser396, and Ser404[52]. Our finding as well as previous report [24] of Ser262 dephosphorylation is in contrast to the increasing phosphorylation of other reported sites. Since phosphorylation of a single residue Ser262 has a major effect on the binding ability of tau protein to microtubules [53], the dephosphorylation of Ser262 could improve the tau protein's capability of stabilizing microtubules during the differentiation process. While additional studies are needed to characterize this RA-induced dephosphorylation pathway, these results underscore the potential of RA for ameliorating the consequences of tau dysregulation.

We conclude that tau protein is a critical mediator of BDNF-induced morphological transformation and possibly also of BDNF-mediated neuroprotection. Since BDNF influences neuronal activity, function, and survival throughout life, much further study is warranted to examine BDNF-tau interactions in sporadic, genetic, and age-related neurodegenerative diseases. Development of transgenic animal models would greatly facilitate such studies.
